# 1274. Implementation of Pharmacist-led Medication Reconciliation in Older PLHIV

**DOI:** 10.1093/ofid/ofac492.1105

**Published:** 2022-12-15

**Authors:** Maile Y Karris, Peter J Mazonson, Lucas Hill, Jeffrey Yin, Kari Abulhosn, Emily Huang, Jeff Berko

**Affiliations:** UC San Diego Medical Center, San Diego, California; Enhanced Health, Inc., Palo Alto, California; University of California, San Diego, San Diego, California; University of California, San Diego, San Diego, California; UCSD, San Diego, California; Enhanced Health, Inc., Palo Alto, California; Enhanced Health, Inc., Palo Alto, California

## Abstract

**Background:**

Older (age ≥ 50) people living with HIV (PLHIV) have a higher prevalence of non-AIDS associated comorbidities and polypharmacy. Optimal care requires accurate knowledge of the number and types of medications older PLHIV take. Most providers use the electronic health record (EHR) to obtain this information, despite reported inaccuracy in other cohorts. Recent efforts have focused on engaging pharmacists to assist with EHR medication reconciliation. This abstract describes the results of a pilot study of phone-based, pharmacist-driven EHR medication reconciliation.

**Methods:**

Older PLHIV reported via an online questionnaire the number of prescription medications they took, with response options ranging from “0” to “11 or more.” Then, a clinic-based pharmacist called each participant to review the daily and prn prescription medications they were taking. Finally, a physician reviewed EHR pharmacy data to count the number of daily and prn prescription medications listed. Wilcoxon rank sum tests compared the medians between the phone review, the self-reported questionnaire, and the EHR review.

**Results:**

Forty participants were offered the intervention, and 26 (65%) were reachable by phone and agreed. Of those, the mean age was 61 years (range 51-73), 89% were male, 67% were White, and the mean number of self-reported comorbid conditions was 11.7 (SD=7.7). In 25/26 (96%) cases, both the number and type of HIV medications in the EHR matched what the pharmacist verified on the phone call. Median number of non-HIV medications are reported in Table 1. Assuming that pharmacist phone calls provided the most accurate data, the EHR overestimated the number of non-HIV medications by 5 medications (P = 0.01), whereas patients’ self-report underestimated by 1.5 medications (P < 0.01).

**Figure:**
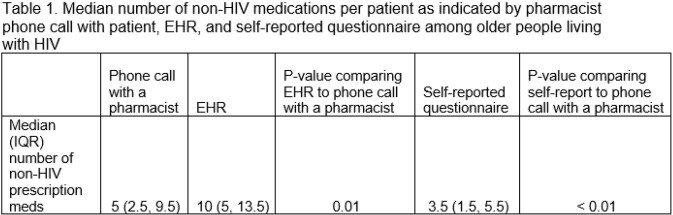

**Conclusion:**

Among older PLHIV, EHR pharmacy data significantly overestimated, and patient self-report significantly underestimated, the number of non-HIV prescription medications. Phone-based, pharmacy-driven medication reconciliation improved upon both EHR-based and self-reported medication accuracy. Based on these findings, future interventions designed to decrease polypharmacy in older PLWHIV can rely upon pharmacist-led, phone-based outreach to ensure that baseline medication information is accurate.

**Disclosures:**

**Maile Y. Karris, MD**, Gilead Sciences: Grant/Research Support|Gilead Sciences: I have received honorarium to speak at conferences|ViiV Healthcare: Grant/Research Support **Peter J. Mazonson, MD, MBA**, ViiV Healthcare: Grant/Research Support **Lucas Hill, PharmD, AAHIVP**, Gilead Sciences: Speakers Bureau **Emily Huang, MPH**, ViiV Healthcare: Grant/Research Support **Jeff Berko, MPH**, ViiV Healthcare: Grant/Research Support.

